# Numerical comparative study of five currently used implants for high tibial osteotomy: realistic loading including muscle forces versus simplified experimental loading

**DOI:** 10.1186/s40634-018-0144-6

**Published:** 2018-08-08

**Authors:** Arnaud Diffo Kaze, Stefan Maas, Slawomir Kedziora, James Belsey, Alexander Haupert, Claude Wolf, Alexander Hoffmann, Dietrich Pape

**Affiliations:** 1 Faculty of Science, Technology and Communication, University of Luxembourg, 6, rue R. Coudenhove-Kalergi, L-1359 Luxembourg, Luxembourg; 2 Department of Orthopedic Surgery, Centre Hospitalier de Luxembourg, L-1460 Luxembourg, Luxembourg; 3 Luxembourg Institute of Health, Luxembourg, L-1445 Luxembourg; 4 Cartilage Net of the Greater Region, 66421 Homburg, Germany; 5 University of Winchester & Basingstoke and North Hampshire Hospital, Sparkford Road, Winchester, SO22 4NR Hampshire England; 6 Saarland University Medical Center, Kirrberger Str., Homburg, 66421 Homburg Germany

**Keywords:** Finite element, Musculoskeletal model, Lower limb, Knee joint, Muscle forces, Stance phase, Medial open wedge HTO, iBalance, Contour lock, PEEKPower, TomoFix, Section forces

## Abstract

**Background:**

Many different fixation devices are used to maintain the correction angle after medial open wedge high tibial osteotomy (MOWHTO). Each device must provide at least sufficient mechanical stability to avoid loss of correction and unwanted fracture of the contralateral cortex until the bone heals. In the present study, the mechanical stability of following different implants was compared: the TomoFix small stature (sm), the TomoFix standard (std), the Contour Lock, the iBalance and the second generation PEEKPower. Simplified loading, usually consisting of a vertical load applied to the tibia plateau, is used for experimental testing of fixation devices and also in numerical studies. Therefore, this study additionally compared this simplified experimental loading with a more realistic loading that includes the muscle forces.

**Method:**

Two types of finite element models, according to the considered loading, were created. The first type numerically simulated the static tests of MOWHTO implants performed in a previous experimental biomechanical study, by applying a vertical compressive load perpendicularly to the plateau of the osteotomized tibia. The second type included muscle forces in finite element models of the lower limb with osteotomized tibiae and simulated the stance phase of normal gait. Section forces in the models were determined and compared. Stresses in the implants and contralateral cortex, and micromovements of the osteotomy wedge, were calculated.

**Results:**

For both loading types, the stresses in the implants were lower than the threshold values defined by the material strength. The stresses in the lateral cortex were smaller than the ultimate tensile strength of the cortical bone. The implants iBalance and Contour Lock allowed the smallest micromovements of the wedge, while the PEEKPower allowed the highest. There was a correlation between the micromovements of the wedge, obtained for the simplified loading of the tibia, and the more realistic loading of the lower limb at 15% of the gait cycle (Pearson’s value *r* = 0.982).

**Conclusions:**

An axial compressive load applied perpendicularly to the tibia plateau, with a magnitude equal to the first peak value of the knee joint contact forces, corresponds quite well to a realistic loading of the tibia during the stance phase of normal gait (at 15% of the gait cycle and a knee flexion of about 22 degrees). However, this magnitude of the knee joint contact forces overloads the tibia compared to more realistic calculations, where the muscle forces are considered. The iBalance and Contour Lock implants provide higher rigidity to the bone-implant constructs compared to the TomoFix and the PEEKPower plates.

## Background

Medial Open Wedge High Tibial Osteotomy (MOWHTO) is a well-established surgery technique used to treat medial compartment osteoarthritis with varus malalignment in young and active patients (Pape et al. 2004). There are many different implants used during medial open wedge high tibial osteotomy (MOWHTO) for correcting varus deformity of the knee associated with medial compartment osteoarthritis. Numerous biomechanical studies have been carried out in order to investigate the mechanical stability of these different fixation systems. Biomechanical studies in orthopaedics can be divided in two groups: experimental and numerical studies. The experimental studies use specimen, which consist of physical prototypes of the bones and cadaveric bones. The numerical studies use three-dimensional (3D) geometries of the bones in finite element models. Finite element models are computer aided designed models that are based on finite element methods. In experimental studies, the loads are applied to the specimen by means of testing machines and the deformations of the specimens are captured by means of sensors. In finite element models, numerical simulations are performed, the loads are applied to the body of interest and the body response to those loads is numerically calculated. These bodies can be bones, soft tissues, implants or screws etc. The response to the applied external loads are deformations, strains and stresses. The biomechanical studies that were carried out in order to investigate the mechanical stability were generally performed experimentally (Spahn and Wittig 2002; Stoffel et al. 2004; Zhim et al. 2005; Agneskirchner et al. 2006; Maas et al. 2013; Watanabe et al. 2014; Diffo Kaze et al. 2015) or by numerical computation, for instance, finite element (FE) analyses (Izaham et al. 2012; Luo et al. 2013; Pauchard et al. 2015; Luo et al. 2015). For the experimental studies, simplified loading of the specimens was applied. Static or dynamic loading conditions consisting generally of axial compression loading (Spahn and Wittig 2002; Stoffel et al. 2004; Zhim et al. 2005; Spahn et al. 2007; Agneskirchner et al. 2006; Maas et al. 2013), or more rarely, of torsional loading (Stoffel et al. 2004) of the specimens are considered. The same applies to numerical studies using FE methods, where the loading conditions were also reduced to axial forces that were applied to the tibia head (Blecha et al. 2005; Izaham et al. 2012; Luo et al. 2013; Pauchard et al. 2015; Luo et al. 2015). In addition to gait loads, Blecha et al. (Blecha et al. 2005) considered femorotibial contact loads due to muscular tonus that compressed the wedge. With the exception of the study by Blecha et al., all the other previously mentioned studies did not consider muscle forces. Experimentally determined knee contact forces are predominantly vertical, though the components in the transverse plane are not zero (Heinlein et al. 2009; Kutzner et al. 2010). Hence, they should be somehow considered. Furthermore, experimental testing procedures should consider realistic loading (Brinkman et al. 2014). However, it remains difficult to realise life-like test conditions in an experimental setting.

The main aim of the present study was to compare five implants currently used for MOWHTO on one side, and to highlight the influence of muscle forces on the other side. For this purpose, two different loading conditions were compared: (1) a simplified loading consisting in applying a vertical compressive force to the tibia plateau, and (2) a more realistic loading considering the muscle forces during the stance phase of normal gait. Furthermore, the study also aimed to match the two loading types, in order to validate the experimental setting, which was used for previous comparative biomechanical experimental studies conducted by Diffo Kaze et al. (Diffo Kaze et al. 2015). Therefore, the simplified and the more complex loading were applied to FE models.

**Fig. 1:**
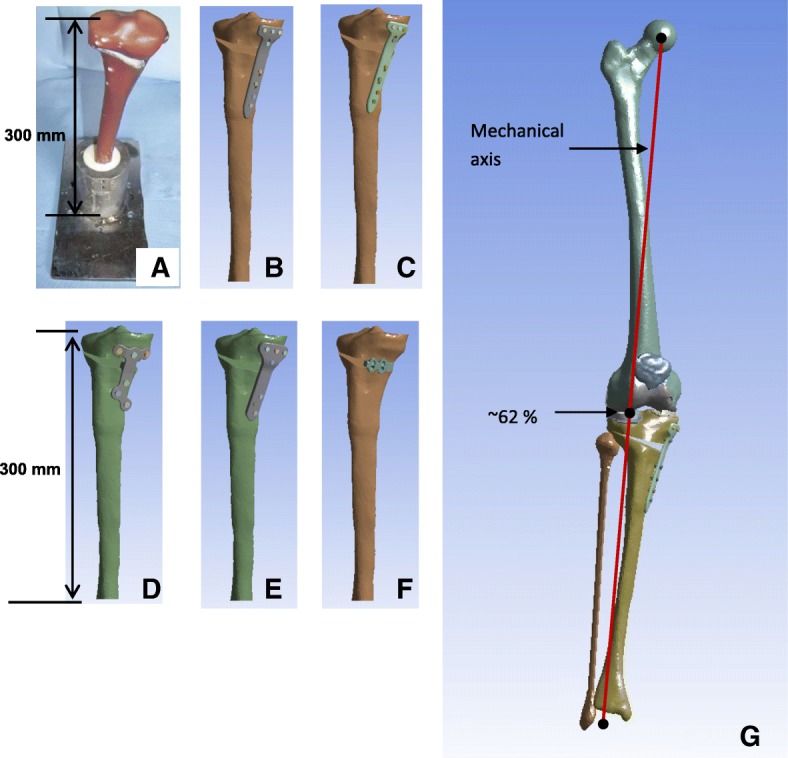
3D Geometries of models: **a** Photo of a tested specimen of the experimental study (Diffo Kaze et al. 2015). 3D geometries of the specimen (**b**) TomoFix Std, (**c**) TomoFix sm, (**d**) Contour Lock, (**e**) PEEKPower, (**f**) iBalance. (**g**) 3D geometries of the lower limb model including the osteotomized tibia with the TomoFix std plate. The mechanical axis passed through the Fujisawa point laterally at 62% of the tibia plateau width

The following five plates were compared (Fig. 1): The Contour Lock, the PEEKPower and the iBalance implants of Arthrex (Arthrex, Munich, Germany), and the TomoFix standard (std) and small stature (sm) plates of Synthes (Depuy Synthes GmbH, Oberdorf, Switzerland). The study compared the effects of the previously mentioned implants in terms of stresses and wedge motions. The hypothesis was that the applied simplified experimental loading corresponds approximately to a realistic loading of the knee joint during the stance phase of gait.

## Methods

### General study design

The simplified loading was applied to FE models simulating the previously performed experimental studies by Diffo Kaze et al. (Diffo Kaze et al. 2015). The more realistic loading was applied to FE models of the lower limb, which were created to achieve the aims of the present study. The results of both FE model types were analysed and compared.

### Bone and plate models

3D geometries of the bones were generated from the mesh of a previous study. This mesh was developed using the state-of-art procedure of 3D geometry acquisition, which was based on segmentation of medical image data and was previously described by Beilas et al. (Beillas et al. 2001). The data for the procedure were collected using medical computer tomography (CT) scanning and magnetic resonance imaging (MRI) on a subject close to a 50th percentile male (Beillas et al. 2001). The FE software package HyperWorks-Radioss (Altair Engineering, Inc., Antony, France) was used to generate the geometries of the bones from the existing mesh and to manually create the geometries of the soft tissues based on anatomy books. The geometry data files were then imported into the Design Modeler of the Release 16.2 of ANSYS Workbench FE software package (Ansys, Inc., Canonsburg, Pennsylvania, U.S.A), which was used to perform the FE analyses. The bones were constituted only of cortical bone and no medullary canal was modelled for the sake of simplicity. The osteotomized tibia was created by: (1) performing an upward mediolateral cut, starting on the medial side at 35 mm distal to the tibia plateau and ending in the contralateral cortical bone at 20 mm distal to the tibia plateau, about 6 mm from the lateral cortex; and (2) by creating an opening of 10° that corresponded to the average value of the correction angle currently performed.

The geometries of the plates and screws (Fig. 1) were created using the software MSC Marc (MSC Software Paris, Paris, France). The screws were modelled as cylindrical with diameters corresponding to the nominal diameters of the device screws following the recommendations of the manufacturers. All parts of the models were meshed with 10 node solid tetrahedral elements.

### Geometries of the models of the biomechanical experimental study

The osteotomized tibia was cut 300 mm distal from the tibia plateau as indicated in our previously performed biomechanical experimental studies (Maas et al. 2013; Diffo Kaze et al. 2015). The cut of the tibia was such that the tibia plateau was horizontal (Fig. 1). Except the fact that uniplanar osteotomies were modelled, all the numerical models were created in conformity with the respective techniques of the fixation systems.

### Geometries of the models of the lower limb

The mechanical axis of the lower limb model passed through the Fujisawa point located laterally at 62% of the tibia plateau width of the osteotomized tibia (Fig. 1g). The lower limb model was constituted of the femur, the tibia, the fibula and the patella. The soft tissues within the knee were the articular cartilages of the tibia and femoral heads, the articular cartilage of the patella, and the menisci and the patellar tendon, which was modelled with three springs. The other knee joint ligaments were not considered. This was justified by the fact that the interface surfaces within the knee joint were modelled as bonded to keep the model linear.

### Material parameters

The constitutive laws for the cortical bones, the soft tissues and the implants were assumed to be linear elastic, homogeneous and isotropic. Young’s modulus of wet embalmed cortical bone of the tibia from younger (41.5 years old) and older (72 years old) men are 18,900 MPa and 16,200 MPa respectively (Evans 1976). Hence a Young’s modulus of 17,000 MPa for the cortical bone was considered for the bones. The biphasic nature of the soft tissues was not taken into account. Furthermore, considering the short loading time during normal walking compared to the viscoelastic time constant of cartilage, the articular cartilage can be modelled as isotropic linear elastic (Donahue et al. 2002; Kiapour et al. 2014). Young’s modulus was 15 MPa for the articular cartilage (Donahue et al. 2002; Hao et al. 2007; Kiapour et al. 2014). Young’s modulus of the menisci is higher in the circumferential direction (120 MPa) compared in radial and transversal directions (20 MPa) (Farrokhi et al. 2011). Hence a Young’s modulus of 120 MPa was considered to model the material behaviour of the menisci as linear isotropic. In the literature, when the soft tissues were considered as homogenous linear isotropic materials, Poisson’s ratio ranged from 0.3 to 0.49 for the menisci, and from 0.4 to 0.475 for the articular cartilage (Beillas et al. 2001; Peña et al. 2005; Hao et al. 2007; Farrokhi et al. 2011). In the present study, Poisson’s ratios were 0.45 for menisci and articular cartilage, and 0.3 for bone.

The patellar tendon was modelled with three springs and the stiffness was determined by using the following formula

$$ k=\frac{E.A}{L}, $$

where E was the Young’s modulus, A the surface of the transversal section and L the length of the patellar tendon. The following values were used: E = 900 MPa (Haraldsson et al. 2005; DeFrate et al. 2007) and A = 160 mm^2^ (Hansen et al. 2006; DeFrate et al. 2007). For the length L of the tendon, a mean value of 5 mm was defined according to the geometry. Hence the stiffness coefficient of the patellar tendon was k = 2880 N/mm, which corresponded to k_spring_ = 960 N/mm for each of the three springs.

The material parameters that were used for the different fixation devices are summarized in Table 1. For the titanium plates, namely the Contour Lock and the TomoFix plates, material properties of the grade II titanium were used in the present study (Thielen 2009; Fischer et al. 2011). The iBalance implant is made of polyetheretherketone (PEEK) and the ultimate tensile strength of the unfilled PEEK ranges between 92 MPa and 100 MPa (MakeItFrom.com 2015). Its other mechanical properties were retrieved from different literature (Kohn and Ducheyne 2005; Garcia-Gonzalez et al. 2015). The PEEKPower plate is made of carbon reinforced PEEK. Its material properties, which were used in the present study, were taken from Fischer et al. (Fischer et al. 2011), except for the fatigue limit and the tensile yield strength. Based on published data of 30% carbon reinforced PEEK 450G (Victrex Ltd 2015), the tensile stress-strain curves of PEEK typically do not exhibit a yield point and its fatigue limit was set at 160 MPa. The flexural yield strength of 30% carbon reinforced PEEK was used instead of its tensile yield strength. The flexural yield strength of 30% carbon reinforced PEEK was 177.5 MPa (Advanced Industrial 2015). Stainless steel was considered for the screws made of steel. The fatigue limit of stainless steel was approximated with 0.4xRm.

**Table 1:** Material parameters of the fixation devices. They were all retrieved from the literature

Material	Young’s modulus (MPa)	Poisson’s ratio *υ*	Ultimate tensile strength *R*_*m*_ (MPa)	Yield strength *R*_*e*_ (MPa)	Fatigue limit *σ*_*D*_ (MPa)
Titanium	110,000	0.3	500	250	200
30% carbon reinforced PEEK	13,000	0.3	210	177.5	160
PEEK	3600	0.38	92–100	92	70
Stainless steel (316 L)	210,000	0.3	480	170	0.4 x *R*_*m*_ =192

### Loading, boundary conditions and analyses

**Fig. 2:**
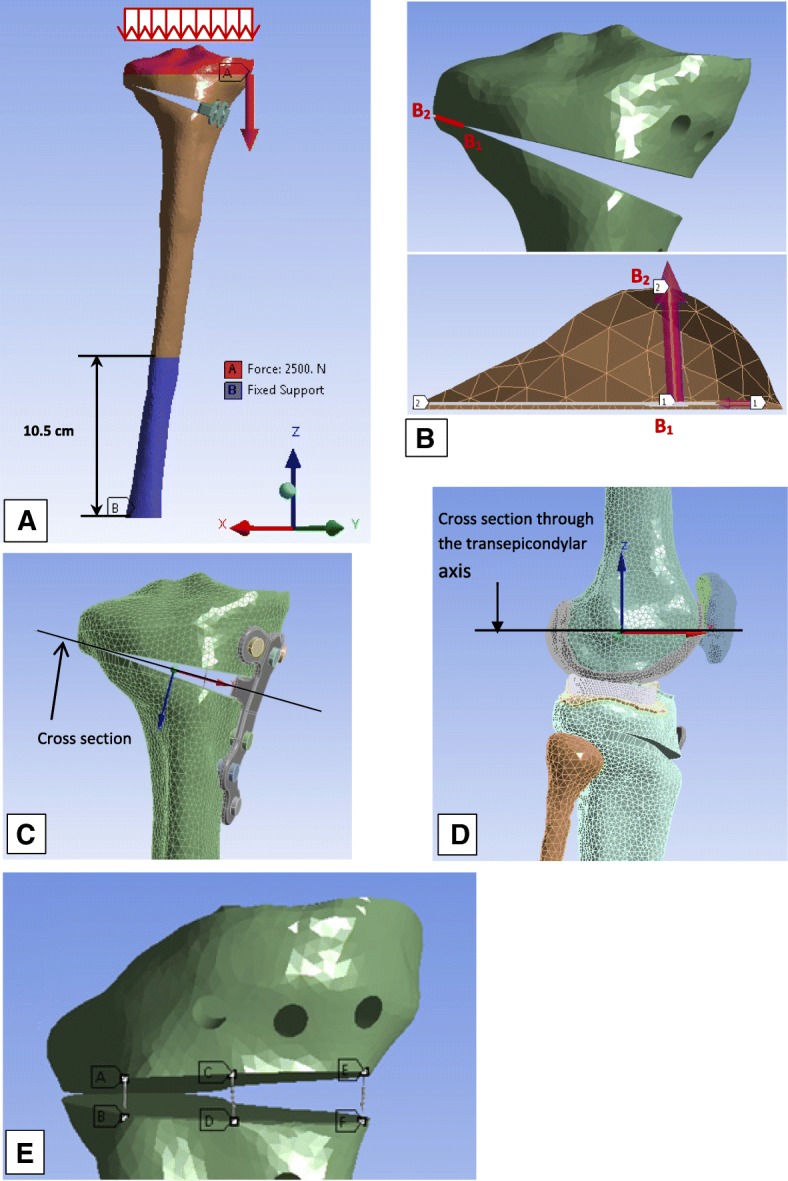
Loading, boundary conditions and analyses: **a** Loading and boundary conditions of the simplified loading models. **b** Cross section of the contralateral cortex and path to check the stresses in the contralateral cortex; B1 and B2 were the starting point and the ending point of the path B1B2 that was chosen and on which the stress distribution was determined. **c** Cross section where section forces and moments were determined in order to highlight the influence of muscle forces. **d** Cross section passing through the transepicondylar axis that was defined in order to calculate the section forces in the knee joint. **e** The three weak springs (**ab, cd** and **ef**) that were used to quantify the micromovements of the wedge

Each of the surfaces’ fractions, which were in contact at the interfaces bone-cartilage, menisci-cartilage, femoral cartilage-patellar cartilage, fixation device-screws, fixation device-bone and bone-screw, were bonded in order to keep the different models linear and reduce complexity. Static analyses were performed. The stresses in the implants and in the contralateral cortical bone, as well as the micromovements of the osteotomized tibia wedge, were determined and analysed. A cross section of the contralateral cortex and a path (B1B2) on that cross section were chosen. The stress distribution on this cross section and on this path was determined. The cross section started at the tip of the wedge and ended at the external margin (Fig. 2**-**b). A suitable amount of micromovement between two fractured bony segments is said to promote callus formation and callus massage, thus helping the fracture healing process (Claes et al. 1997, 1998; Claes and Heigele 1999; Yamaji et al. 2001; Klein et al. 2003). The micromovements were assessed by the length variation of three very weak springs (Stiffness: 10^− 3^ N/m) linking the outermost distal and proximal borders of the wedge on the medial side (Fig. 2**-**e**)**. Influence of the muscle forces was determined by comparing the section forces on cross sections (Fig. 2-c and d) of the models for the two different loading types.

#### Loading and boundary conditions for the simulation of the stance phase of normal gait including the muscle forces

**Fig. 3:**
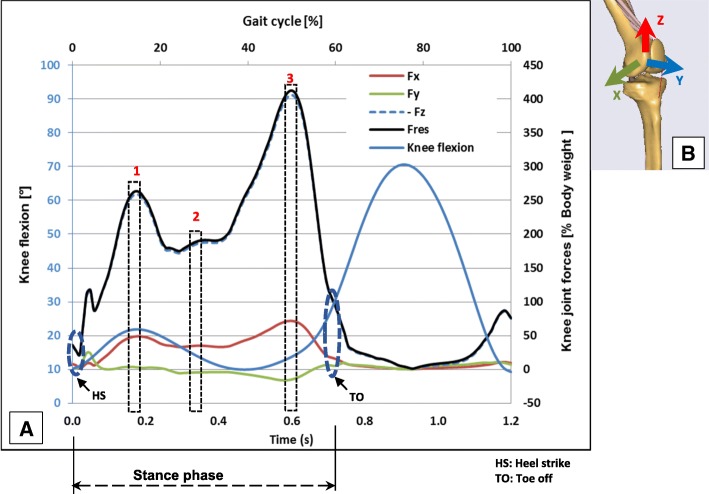
The three analysed positions: **a** Components of the knee joint forces and knee joint flexion angle over the complete gait cycle. **b** Coordinate system used to define the calculated knee joint forces

**Fig. 4:**
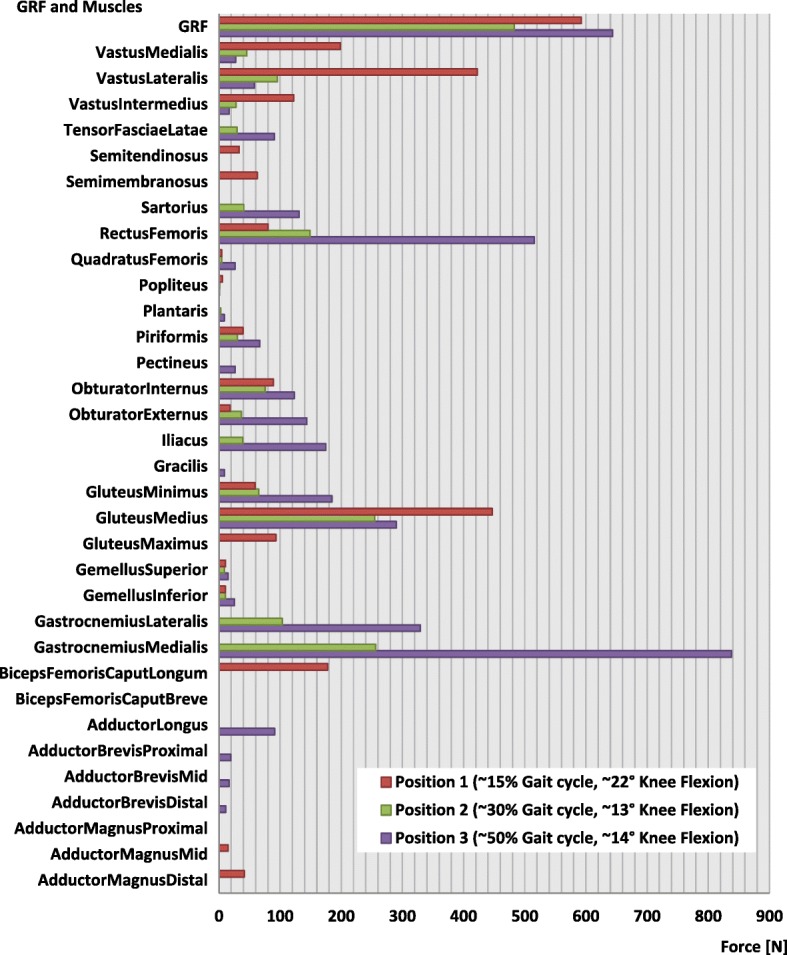
Magnitudes of the muscle forces at the three selected positions: The set of the active muscles is different from one position to another

Inertia effects are negligible in comparison to muscle forces during normal walking and hence were not considered in the present study. A more detailed description of the modelling methods of the present finite element models of the lower limb was published in a previous study (Diffo Kaze et al. 2017**)**. The muscle forces acting in the lower limb during normal gait were calculated by means of a validated musculoskeletal rigid body (MRB) model (Manders et al. 2008**;** Thielen 2009; Asfour and Eltoukhy 2011) This MRB model, namely the Gaitfullbody model, is available in the model repository of the musculoskeletal modelling software AnyBody version 6 (AnyBody Technology 2014). The knee joint in the MRB was modelled as a revolute joint that was located in the middle of the transepicondylar axis. Hence, section forces in the cross section of the femoral head of the FE models passing through the transepicondylar axis (Fig. 2**-**d) were determined and compared to the knee joint forces, which were calculated by means of the MRB model. In order to simulate the stance phase, three positions of the stance phase were selected (Fig. 3) based on the knee joint forces that were calculated using the MRB model. The selected positions represented three different loading configurations that corresponded to the first peak (positions 1: ~ 15% gait cycle and 22° knee flexion), a local minimum between the two peaks (positions 2: ~ 30% gait cycle and 13° knee flexion) and the second peak of the knee joint force (positions 3: ~ 50% gait cycle and 14° knee flexion). The foot was not modelled, and a segment was used to model the foot sole and to locate the centre of pressure (COP), to which the ground reaction forces (GRF) were applied. The experimentally measured knee contact forces by Bergmann et al. (Bergmann 2008) were comparable to the knee joint forces of the used MRB model (Diffo Kaze et al. 2017). Hence, the muscle forces calculated by using the MRB model (Fig. 4) and the GRF were assumed to be valid. These data were then exported from AnyBody and were applied to the finite element models of the lower limb with the osteotomized tibia and the different implants (Fig. 1**-**g). The femur was attached to the ground with three very stiff springs (Stiffness: 10^9^ N/m), oriented in all three spatial directions, in order to constrain the translational degrees of freedom (DOF) of the centre of the femoral head. Three weak stabilisation springs (Stiffness: 1 N/mm), which were oriented in the three spatial directions, were attached at the distal basis of the tibia in order to avoid numerical instability of the models. The muscle forces were applied as distributed load over their respective origin and insertion areas, which were reproduced on the bone geometry surfaces, according to their anatomical locations. Tibia and fibula were bonded at their common interface.

#### Loading and boundary conditions for the simulation of the experiments

The kinematics and kinetics data of a subject with a mass of 62 kg were used for the MRB model. The calculated maximal axial compressive load on the knee joint was 410% body weight (BW). For a person with a BW of 608.22 N, 410% BW corresponds to 2500 N, which was applied to the tibia head of the specimen, while the distal end was fixed (Fig. 2-a**)**.

## Results

### Comparison of the loadings

The section forces and moments in the cross section following the wedge as shown in Fig. 2**-c** are given in Table 2**.** The forces and the moments were given in the local coordinate system located at the centroid of the cross section. The section forces, due to the muscles, were smaller than those resulting from the applied vertical load of 2500 N, which considered as being a simplified loading. The resultant moments were of the same order of magnitude. There were no linear correlations between the loading types regarding the components of the section forces and moments. The resultant of the section force in the case of the simplified loading (2496 N) was approximately double the resultant of the section force (1251 N) in the case of muscle forces acting on the lower limb in position 1; and, both section forces were oriented distally and posteriorly.

**Table 2:** Comparison of the section forces and moments in the cross section shown in Fig. 2-C. The forces and the moments were given in the local coordinate system located at the centroid of the cross section. Fx, Fy, Fz were the components of the Force; Mx, My, Mz were the components of the Moment. Fres and Mres were the resulting of the force and of the moment respectively

Loading variations		Mean values of the section forces							
		Force [N]				Moment [N.m]			
		Fx	Fy	Fz	Fres	Mx	My	Mz	Mres
Simplified loading		797	−20	2365	2496	−17	−23	6	28
Muscle actions	Position 1	274	−7	1220	1251	−11	1	14	18
	Position 2	198	32	792	817	−4	−12	10	16
	Position 3	358	20	1762	1798	−2	−19	12	23

Table 3 gives the components of the section forces in the section passing through the transepicondylar axis of the femur in the FE models including muscle forces and the knee joint forces of the MRB model. The resultants of these section forces were comparable to those of the knee joint forces of the MRB model. The components of the forces were referred to the coordinate system of Fig. 3-b. For the lower limb in position 1, the section force was almost vertical as in the case of the simplified experimental loading. The magnitudes of the section forces in the femoral head were higher than those in the tibia wedge. This was due to the action of the muscles that had their insertions above the cross section of the wedge.

**Table 3:** Comparison of the knee joint force in the MRB model with the section forces in the cross section passing through the transepicondylar axis of the femur (Fig. 2-d). The forces were given in the coordinate system of Fig. 3-b. Fx, Fy, Fz were the components of the Force; Mx, My, Mz were the components of the Moment. Fres and Mres were the resulting of the force and of the moment respectively

Positions simulated	Mean values of the components of the section forces in the cross section passing through the transepicondylar axis of the femur (Fig. 2-d)				Resultant of the knee joint force from the MRB model
	Fx[N]	Fy[N]	Fz[N]	Fres[N]	Fres[N]
Position 1	−7	1790	71	1791	1650
Position 2	609	978	198	1169	1100
Position 3	1284	2275	180	2618	2500

### Stress analysis of the implants

**Fig. 5:**
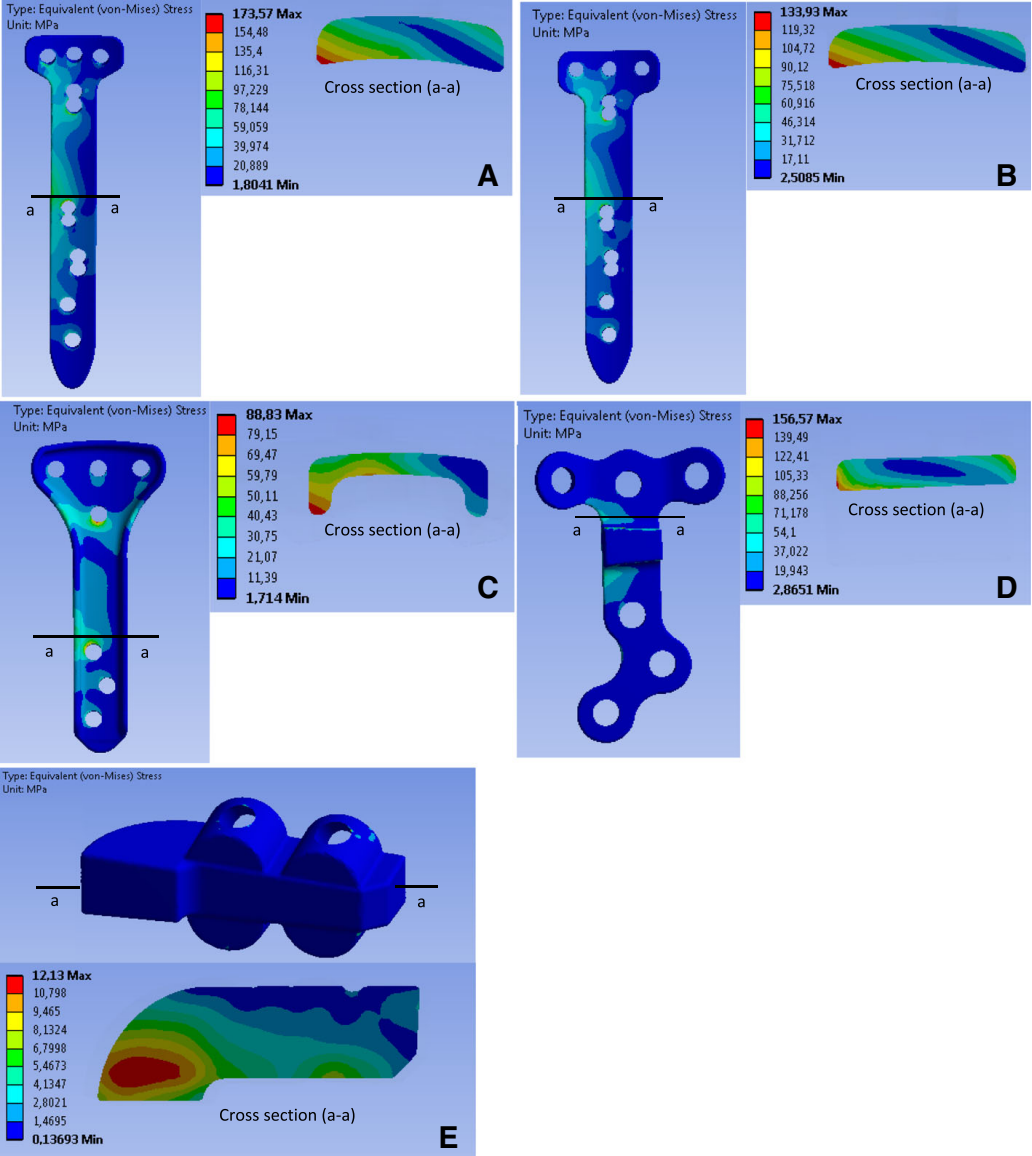
Equivalent stress (von-Mises) in the implants after the simplified loading of the tibia: The letters **a, b, c, d** and **e** indicates the TomoFix sm, the TomoFix std., the PEEKPower, the Contour Lock and the iBalance implants

**Fig. 6:**
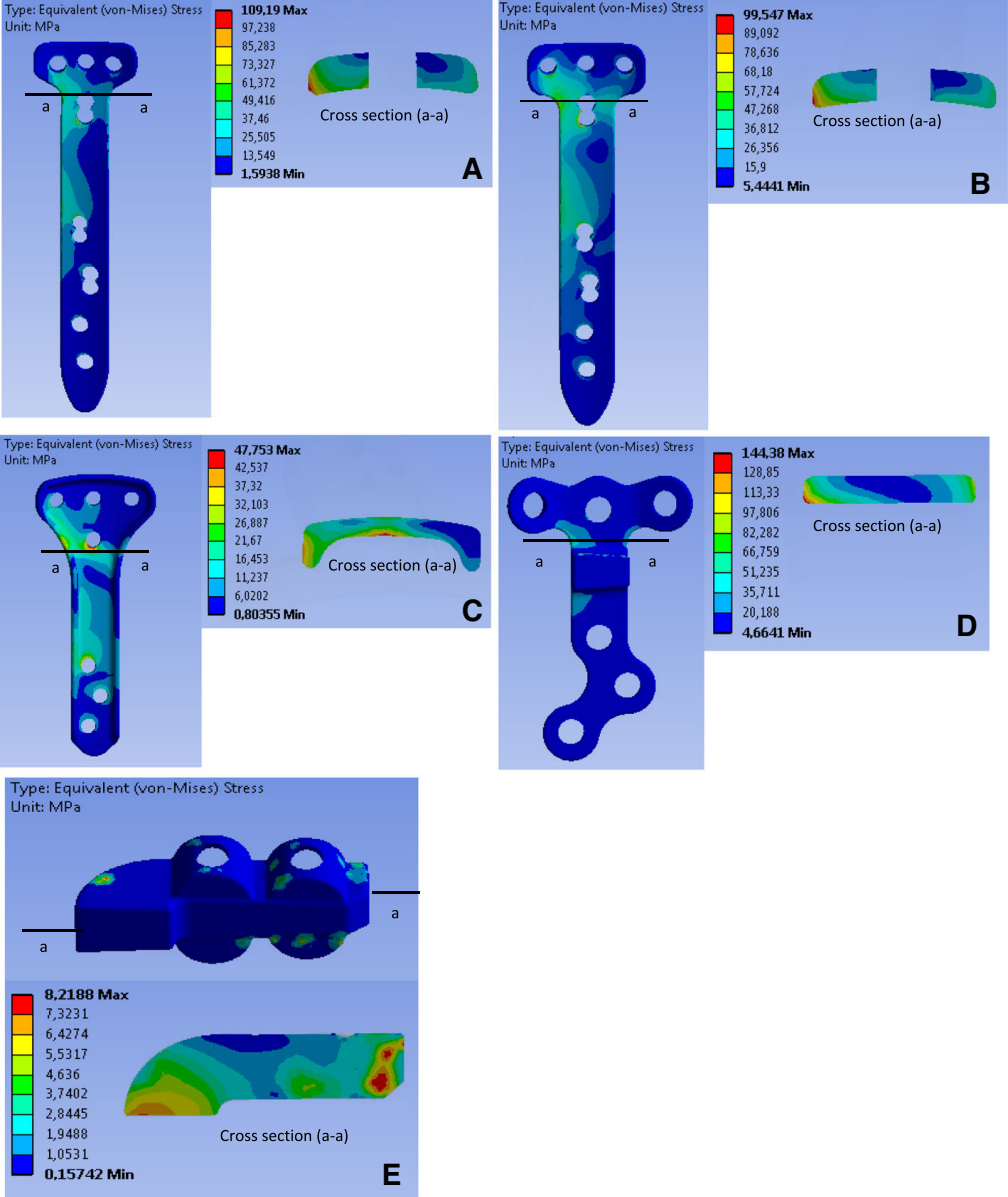
Equivalent stress (von-Mises) in the implants in position 1: The letters **a, b, c, d** and **e** indicate the TomoFix sm, the TomoFix std., the PEEKPower, the Contour Lock and the iBalance implants

**Fig. 7:**
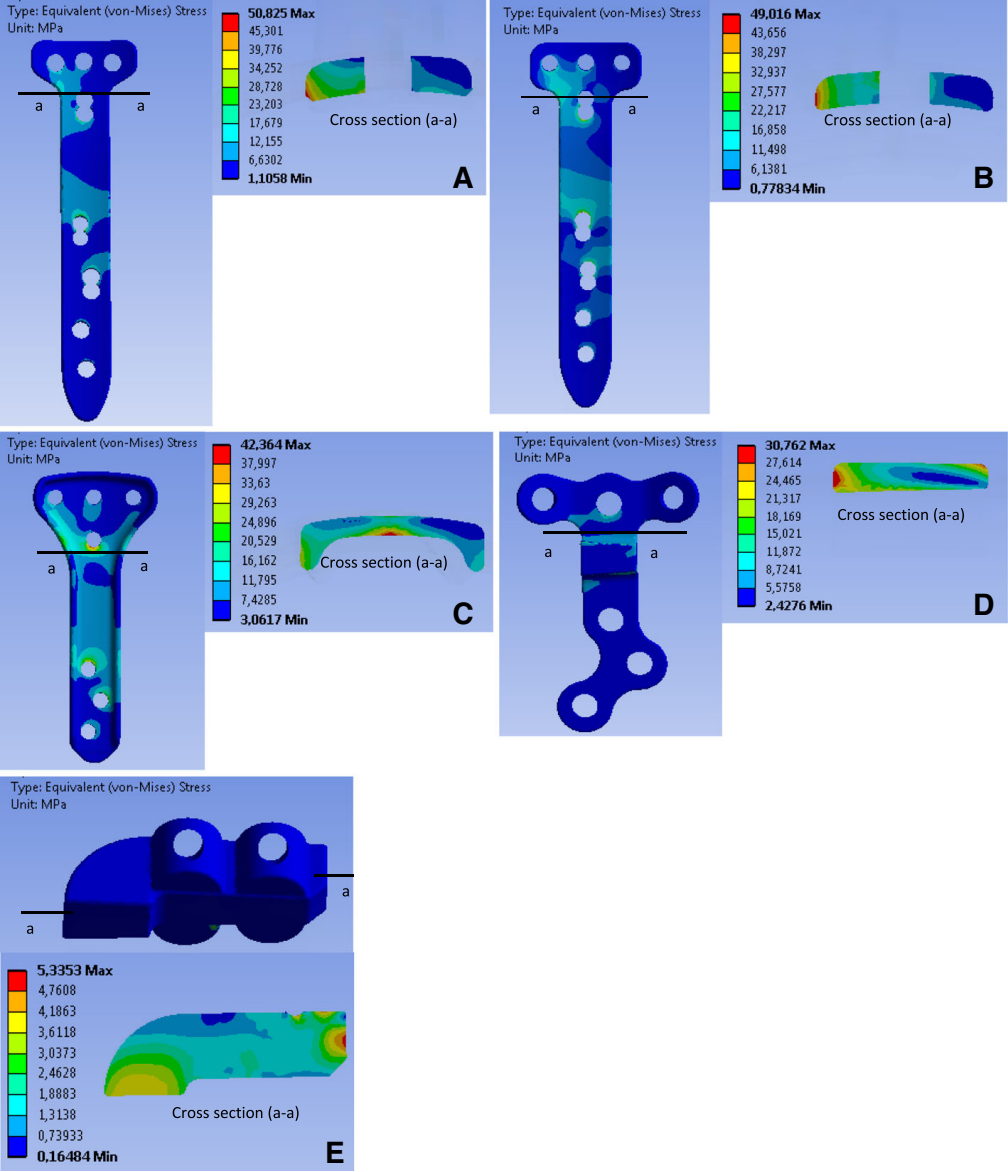
Equivalent stress (von-Mises) in the implants in position 2:.The letters **a, b, c, d** and **e** indicate the TomoFix sm, the TomoFix std., the PEEKPower, the Contour Lock and the iBalance implants

**Fig. 8:**
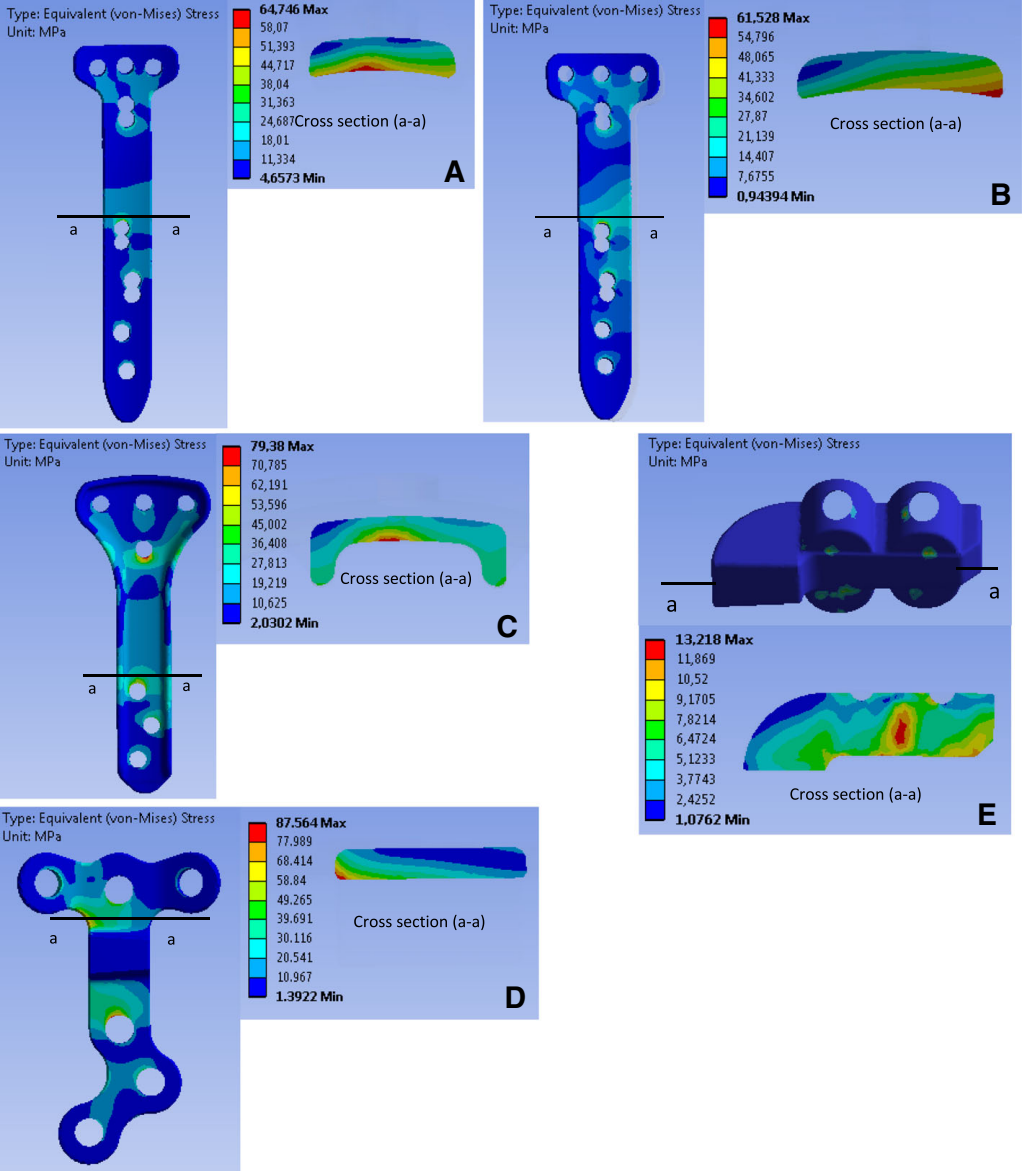
Equivalent stress (von-Mises) in the implants in position 3: The letters **a, b, c, d** and **e** indicate the TomoFix sm, the TomoFix std., the PEEKPower, the Contour Lock and the iBalance implants

A cross section (a-a) was defined in the implant where the equivalent von-Mises stresses were maximal. The stress distribution of this cross section was given for the different implants. Figure 5 shows the equivalent von-Mises stress in the implants after a static compressive load was applied to the tibia plateau, while Fig. 6**,** Fig. 7 and Fig. 8 show the equivalent von-Mises stress after the lower limb was subjected to the muscle forces.

### Case of the simplified loading consisting in applying a vertical compressive load of 2500 N to tibia plateau (Fig. 5)

The maximal von-Mises equivalent stress was located, for the PEEKPower and the two TomoFix plates, in the region situated on the right margin just above the distal holes. The right side of the implants corresponded to the posterior region of the tibia. For the Contour Lock plate, the highly stressed region was located at the plate corner on the right side above the spacer. The stress distribution of the above mentioned plates highlighted a bending for which compressive stresses were higher than tensile stresses. For the iBalance, the highly stressed volume was in the middle on the right side.

### Case of the more realistic loading including the muscle actions (Fig. 6, Fig. 7 and Fig. 8)

Position 1 – Fig. 6: The maximal von-Mises equivalent stress was located at the plate corner on the right side for all the plates except for the iBalance implant. Additionally, for the PEEKPower, the margin of the middle region below the proximal hole, above the wedge, was highly stressed. For the iBalance implant, the highly stressed volume was located in the middle of the implant, but on the left side.

Position 2 – Fig. 7: As in position 1, the maximal von-Mises equivalent stress occurred, for the Contour Lock and the two TomoFix plates, at the corner on the right side. The margin of the middle region of the PEEKPower plate below the proximal hole above the wedge was the hot spot. The maximal stresses were located at the proximal part of the four other implants and in the middle region of the iBalance implant.

Position 3 – Fig. 8**:** In position 3, the maximal equivalent stresses were located for the TomoFix sm and the PEEKPower plates in the margin of the middle region above the first distal hole. For the TomoFix std., the region of maximal stress was located on the right. The stress distribution in the cross section was similar to that of position 2. Stresses were additionally concentrated above the first distal hole. For the iBalance, the most stressed volume was located in the middle of the centre of the implant.

### Maximal stress values in the implants

Table 4 gives a comparative summary of the maximal equivalent stresses (*σ*_*eq*, *max*_) in the implants and the threshold values of the materials. All the equivalent stresses in the implants were smaller than the yield stress *R*_*e*_, the ultimate strength *R*_*m*_ and the fatigue limit *σ*_*D*_.

**Table 4:** Comparative summary of the maximal equivalent von-Mises stresses in the implants and the corresponding material strength values

Implants	*σ*_*eq*, *max*_ (MPa)				*R*_*e*_ (MPa)	*R*_*m*_ (MPa)	*σ*_*D*_ (MPa)
	Simplified loading	Muscle actions					
		Position 1	Position 2	Position 3			
Contour Lock	157	144	31	88	250	500	200
TomoFix sm	174	109	51	65	250	500	200
iBalance	12	8	5	13	92	(92–100)	70
PEEKPower	89	48	42	79	177.5	210	160
TomoFix std	134	100	49	62	250	500	200

By taking the yield strength *R*_*e*_ as the reference threshold, all the implants showed safety factors higher than or equal to 1.4, independent to the load applied. The smallest safety factor (1.4) was obtained for the TomoFix sm in the case of the simplified loading, and the highest safety factor (18.4) was obtained for the iBalance implant at position 2.

### Predominant types of stresses in the implants

The implants were predominantly subjected to bending. The tensile (T) and compressive (C) stresses values in the cross sections of maximum equivalent stress in the implants are reported in Table 5**.**

**Table 5:** Tensile (T) and compressive (C) stresses in the cross sections of maximum von-Mises stress in the implants

Implants	Simplified loading		Muscle actions					
			Position 1		Position 2		Position 3	
	T (MPa)	C (MPa)	T (MPa)	C (MPa)	T (MPa)	C (MPa)	T (MPa)	C (MPa)
Contour Lock	108	−156	81	−137	58	−60	16	−47
TomoFix sm	83	− 174	51	−110	2	−50	18	−68
iBalance	1	−12	7	−8	2	−4	4	−11
PEEKPower	9	−89	24	−46	16	−13	16	−86
TomoFix std	53	−134	41	−82	9	−46	15	−62

The maximal von-Mises stresses were predominantly compressive stresses. The maximal tensile stresses were obtained when the lower limb was in position 1 under muscle actions.

### Stress distributions in the contralateral cortex

**Fig. 9:**
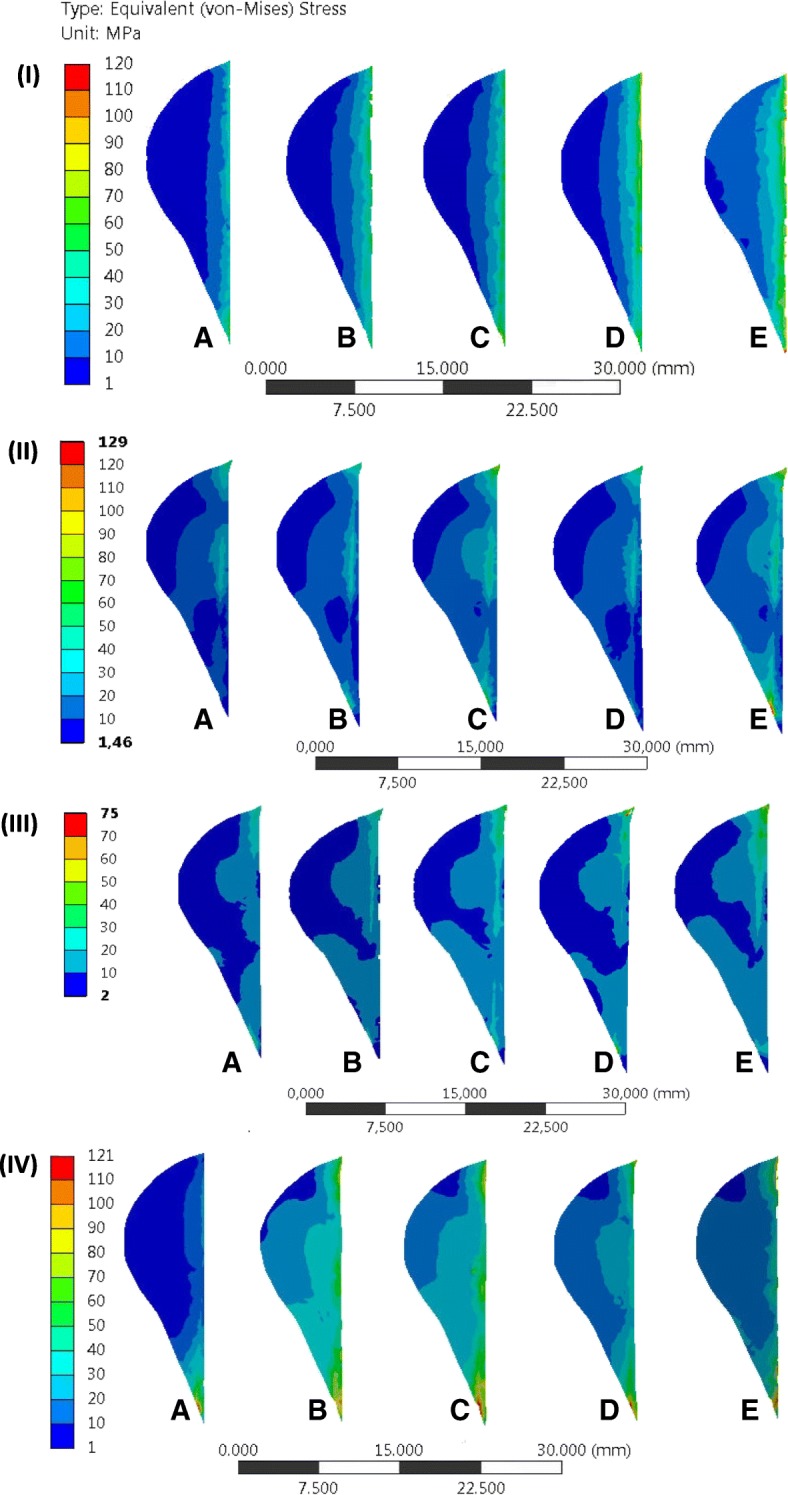
Equivalent (von-Mises) stress in the contralateral cortex: (**I**) simplified loading of the tibia head. (**II**) Lower limb with muscle force loading in position 1. (**III**) Lower limb with muscle force loading in position 2. (**IV**) Lower limb with muscle force loading in position 3. The letters **a, b, c, d** and **e** indicate the iBalance, the TomoFix sm, the TomoFix std., the Contour Lock and the PEEKPower implants

**Fig. 10:**
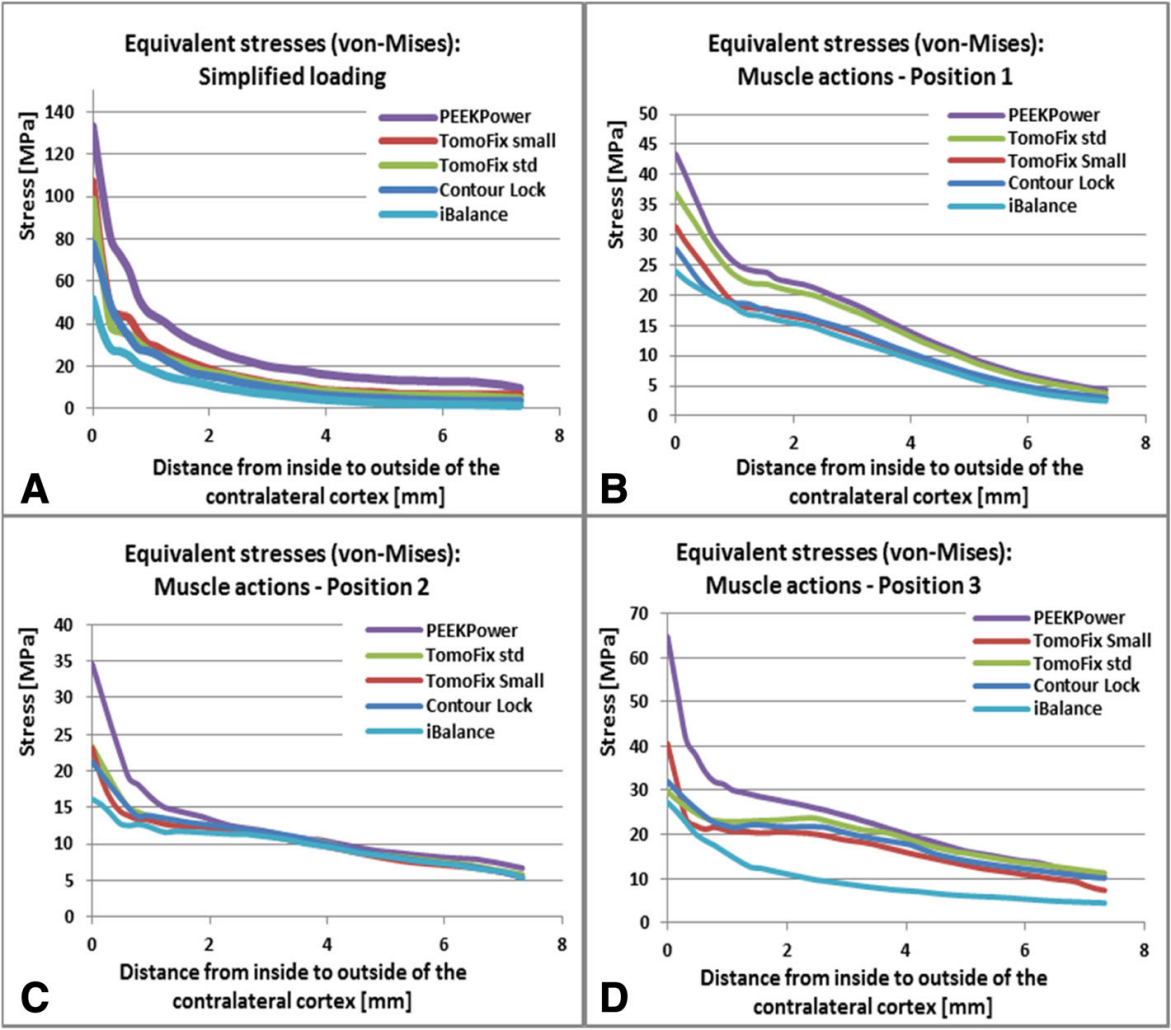
Equivalent stresses along the path B1B2 of the contralateral cortex (Fig. 2**-**b)**: a** simplified loading of the tibia head. **b** Lower limb under muscle force loading in the position 1. **c** Lower limb under muscle force loading in the position 2. **d** Lower limb under muscle force loading in the position 3

Higher stresses in the tibia were located in the contralateral cortical bone. Figure 9 shows the distribution of the equivalent von-Mises stress in the cross section of the contralateral cortex (Fig. 2-b). The stress distribution altered almost uniformly from the lateral to the medial side in the case of the simplified loading (Fig. 9-I). This was not the case for the other loading variations including the muscle forces (Fig. 9-II to IV). The maximal stresses were located in the area at the tip of the open wedge on the anterior and posterior corners of the cross section. Beyond the tip of the open wedge and the corners, the stresses were smaller than 50 MPa (Fig. 9-I to III), except in the case of position 4 (Fig. 9-IV), where they were smaller than 70 MPa. The stresses in the contralateral cortex decreased rapidly from the hot spot to the minimal stresses (Fig. 10). The iBalance implant offered the best support to the contralateral cortex whereas the PEEKPower offered the least, regardless of the considered loading.

Based on the stress analysis along the chosen path B1B2 (Fig. 2-b) in the case of the simplified loading (Fig. 10-a), the Contour Lock plate appeared to relieve the contralateral cortex of high stresses better than the TomoFix plates. In position 1, the TomoFix std. was inferior to the iBalance, the Contour Lock and the TomoFix sm regarding that property. In position 2 and 3, apart from the PEEKPower and the iBalance, the other plates were approximately equivalent.

### Micromovements of the wedge

**Fig. 11:**
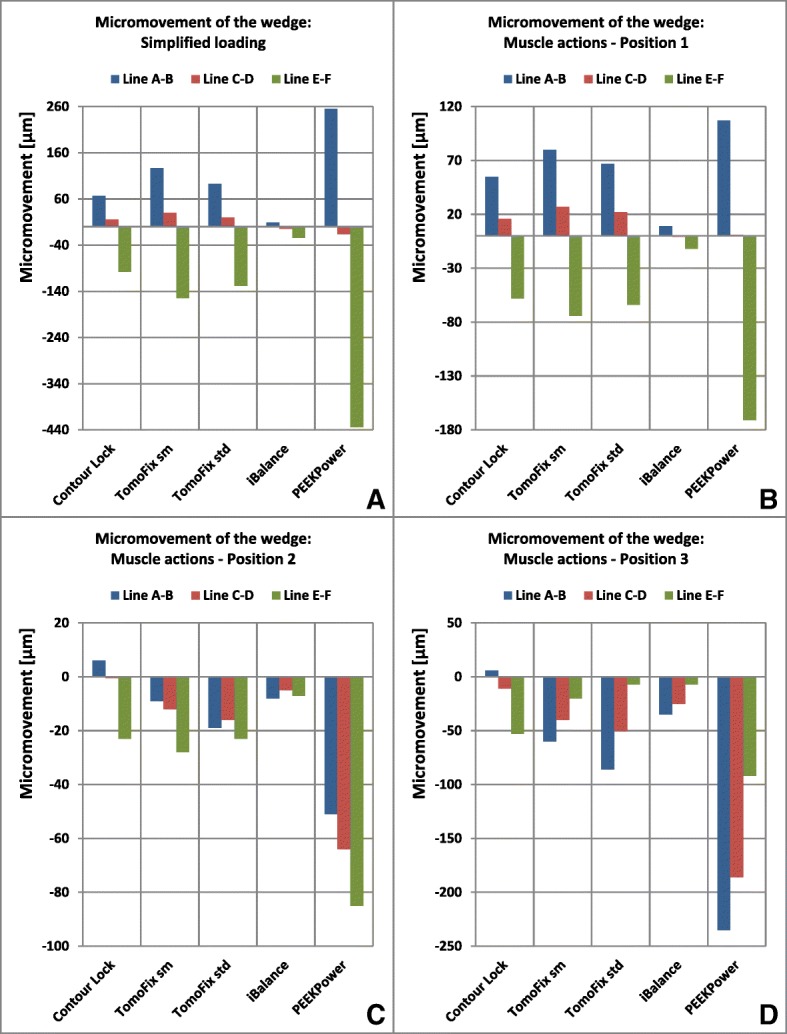
Micromovements of the open wedge: The micromovements under the simplified loading and the muscle force loading in position 1 were correlated

Figure 11 shows the micromovements captured by means of the three weak springs (AB, CD and EF) that were attached between the outermost distal and proximal borders of the wedge on the medial side. The behaviours of the wedge were qualitatively identical under the simplified loading, and under the muscle force loading in position 1 (Fig. 11-a and b). The Pearson’s value was *r* = 0.982. This identical comportment corresponded to a posterior closing and an anterior opening of the wedge. In the previously mentioned comportment, the closing and the opening of the wedge were consistent with a compression and a distraction respectively. There was no other identical deformation pattern of the wedge between the other loading variations. In position 3, for all implants except for the Contour Lock, the wedge exhibited an anterior closing and a posterior opening, although, the latter still resulted in compression of the wedge (Fig. 11-d). The highest micromovements under muscle forces were obtained in position 3 (235 μm for the PEEKPower plate). Generally, the iBalance implant showed the smallest micromovements (7 μm in average), when the muscles forces were included.

## Discussion

The key finding of the present study is that perpendicularly applying a vertical load to the tibia plateau is qualitatively equivalent to the realistic loading of the tibia including muscle forces in position 1 of the stance phase. The position 1 corresponded to 15% of gait cycle with 22° knee flexion. The stresses in all the implants were smaller than the yield stress, the ultimate strength and the fatigue limit of the respective material. This last observation means that the implants will not fail due to fatigue in the modelled biomechanical environment under physiological loading during normal walking. The PEEKPower implant exhibited the highest micromovements of the wedge, whereas the iBalance exhibited the smallest.

Two loading types were considered in the present FE analysis study; the first type consisted of a vertical load being applied perpendicularly on the tibia head. The second loading type consisted of three loading variations of the tibia, including the muscle forces, in three positions of the lower limb in the stance phase. The loading in the first position appeared to be similar to an axial load perpendicular to the tibia plateau. On the other hand, the loading of the lower limb in the other two positions led to different observations compared to the simple vertical loading. This emphasises the necessity to improve the experimental testing by incorporating other forces and creating comparable multiaxial loading of the osteotomized tibia. We did not find any other study analysing the MOWHTO by means of FE analysis, which includes detailed muscle forces.

As reported in Table 5, the stress concentrations predominantly consisted of compressive stresses. The stress concentrations in all the implants were smaller than the tensile strength values of the respective materials, as already mentioned. However, it should be noted that the mechanical properties of the material used in the present study result from tensile tests, and are normally smaller than compressive strength values i.e. the safety is even higher. Except for the iBalance implant, the highest equivalent stresses in the implants resulted from the simplified loading and not from the realistic loading, which included the muscle forces. The preceding observation corresponds to the fact that the section forces in the cross section passing through the middle of the wedge (Fig. 2-c, Table 2) were higher for the simplified loading than for the muscle force loading. Although the section force at the level of the transepicondylar axis corresponded to the knee contact forces from the validated MRB model. This means that the combined action of the muscle forces and the GRF on the tibia plateau is not the same at different locations of the tibia. Hence, applying the peak values of the knee joint contact forces to the tibia plateau in FE analysis including the tibia, as it is commonly performed in the literature (Blecha et al. 2005; Izaham et al. 2012; Luo et al. 2015; Huang et al. 2015), and as it was done for the simplified loading in the present study, overloads the tibia compared to a more realistic loading including the muscle forces.

The stress concentrations in the TomoFix sm and in the TomoFix std. were located at the same part of the plate, but were higher for the TomoFix sm. This is correlated to the fact that the two TomoFix plates have the same design and the TomoFix sm is smaller than the TomoFix std. and was designed for small stature patients. The fact that the stresses were the smallest in the iBalance implant for all the loading variations considered, can be attributed to the fact that this implant has the largest cross section.

The equivalent von-Mises stresses in the contralateral cortex were higher at the tip of the wedge compared to its values in the external side (Fig. 9). For all implant, regardless of the loading considered, the stress concentration on the cross section of the contralateral cortex was smaller than the ultimate stresses of the cortical bone, which were reported to be 163 MPa and 183 MPa in axial tension and compression respectively, by Burstein et al. (Burstein et al. 1976). The iBalance allowed the largest portion of the cross section with stresses lower than 10 MPa and the PEEKPower the smallest. The stress evolution along the chosen path B1B2 (Fig. 2-b) in the case of the simplified loading (Fig. 10-a) led to a similar ranking of the fixation devices as in our previously performed biomechanical experimental study (Diffo Kaze et al. 2015). There we reported that the Contour Lock had the highest mechanical stability followed by the iBalance, the TomoFix std., the TomoFix sm and the PEEKPower. This ranking suggests that the finite element models and the experimental setting were in good agreement.

Micromovements of the wedge were quite small for the iBalance and the Contour Lock implants. This observation is in good agreement with the results of our biomechanical experimental study (Diffo Kaze et al. 2015), where we compared the static and fatigue strength of the implants considered in the present study. Röderer et al. reported results that linked low bone formation underneath locking plates to high implant stiffness in locking plating (Röderer et al. 2014). Therefore, the advantage of the higher mechanical stability provided by the implants iBalance and Contour Lock, in comparison to the PEEKPower and the TomoFix plates, could be called into question. Minimum mechanical stability is a necessary but not sufficient condition for bone formation; there are other aspects that need to be considered (Schröter et al. 2015). We did not find clinical postoperative studies of MOWHTO with the iBalance and Contour Lock implants. Numerous clinical postoperative studies reported good outcomes of MOWHTO in term of stable correction, early full weight bearing and bone union for TomoFix plates (Takeuchi et al. 2009; Brinkman et al. 2010; Brosset et al. 2011, Schröter et al. 2017). Cotic et al. reported that the second generation PEEKPower plate was safe for MOWHTO, although there was one case of non-union out of 28 patients (Cotic et al. 2015).

Luo et al. reported micromovements of the wedge, 420 μm opening versus 926 μm closing, with a TomoFix plate (Luo et al. 2015). However, trabecular bone was considered in their model and the axial load that was applied on the tibia plateau was asymmetrical with a distribution of 40% on the lateral side. The micromovements were very small and the differences observed between the present study and the study by Luo et al. can also be related to differences in Young’s moduli used for the plates. Luo et al. did not indicate which value they used.

In a clinical point of view, the implants iBalance and Contour Lock should be used if a rigid bone-implant construct is required. Flexible bone-implant constructs will be obtained with the TomoFix and PEEKPower plates. However, one should proceed with caution while transferring the results of the present study to clinical settings, because the present study has some limitations. An obvious limitation is the absence of trabecular bone in the modelling of the tibia. The trabecular bone was not considered in order to avoid the modelling of the interface cortical bone-trabecular bone, which is not a well-defined limit. Furthermore, the contact interfaces were modelled as bonded. The jaw compressive force induced by the remaining intact opposite cortex, medial collateral and patellar ligament, which attempt to close the wedge after opening by the surgeon (Blecha et al. 2005), was not considered. The modelled osteotomized tibia corresponded to a state directly after the osteotomy without onset of ossification. The muscle forces that were included in the study were calculated by means of kinetics and kinematics data obtained from an asymptomatic healthy individual. Full weight bearing is not prescribed until at least two weeks after surgery, but was considered in the present study.

## Conclusion

An axial compressive load applied perpendicularly to the tibia plateau, with a magnitude equal to the first peak value of the knee joint contact forces, corresponds quite well to a realistic loading of the tibia during the stance phase of normal gait (at 15% of gait cycle and a knee flexion of about 22 degrees). However, this magnitude of the knee joint contact forces overloads the tibia compared to more realistic calculations, where the muscle forces are considered. The iBalance and Contour Lock implants provide higher rigidity to the bone-implant constructs compared to the TomoFix and the PEEKPower plates.

## Abbreviations

3D: Three-dimensional
BW: body weight
COP: centre of pressure
DOF: degrees of freedom
FE: finite element
GRF: ground reaction forces
MOWHTO: Medial open wedge high tibial osteotomy
MRB: musculoskeletal rigid body
MRI: magnetic resonance imaging
PEEK: polyetheretherketone
sm: Small stature
std: Standard
